# Development of a High-Resolution All-Fiber Homodyne Laser Doppler Vibrometer

**DOI:** 10.3390/s20205801

**Published:** 2020-10-14

**Authors:** Jianhua Shang, Yan He, Qi Wang, Yilun Li, Lihong Ren

**Affiliations:** 1 Shanghai Collaborative Innovation Center for High Performance Fiber Composites, School of Information Science and Technology, Donghua University, Shanghai 201620, China; qwdhu2019@163.com (Q.W.); liyilun@dhu.edu.cn (Y.L.); lhren@dhu.edu.cn (L.R.); 2 Shanghai Institute of Optics and Fine Mechanics, Chinese Academy of Sciences, Shanghai 201800, China; heyan@siom.ac.cn

**Keywords:** LDV, homodyne, velocity resolution, minimum detectable SPL

## Abstract

Based on the homodyne detection, a compact and cost-effective all-fiber laser Doppler vibrometer (LDV) with high resolution is presented. For the signal processing, the discrimination algorithm combined with the nonorthogonal correction is applied. The algorithm corrects the quadrature imbalance and other nonlinearity. In the calibration experiment, with the glass pasted on a piezoceramic transducer (PZT), the velocity resolution of 62 nm/s at 4 kHz and displacement resolution of 2.468 pm are achieved. For the LDV-based acousto-optic communication, the minimum detectable sound pressure level (SPL) reached 0.12 Pa under the hydrostatic air-water surface. The results demonstrate that the designed homodyne LDV has a low system background noise and can offer high precision in the vibration measurement.

## 1. Introduction

The laser Doppler vibrometer (LDV) is adopted primarily to measure the vibration characteristics in an optical nonintrusive and remote way. The principle of the LDV relies on the coherent detection concerning the Doppler frequency shift of the reflected or scattered laser beam from a moving target [1,2]. Hitherto, its development has received great attention, and it is also widely applied to various fields ranging from the acousto-optic detection for the underwater sound [3], the remote voice acquirement [4], the health monitoring for the composite materials [5,6,7,8], the biomedical assessments [9,10,11,12], the trace explosive detection [13,14], and the vibration analysis of the component motions and the civil structures [15,16]. Compared with the contact sensors, such as the piezoelectric accelerometer, the LDV is good at performing the noncontact vibration measurement of the rotating structures. Abbas et al. [17] obtained the underwater vibration response of the blades of rotating propellers by means of the LDV and compared this response with that obtained with the wireless contact piezoelectric accelerometers.

Concerning whether the intermediate frequency (IF) equals zero or not, the LDV can be classified into the homodyne detection and heterodyne detection. Pursuing a higher phase-sensitive detection, some consideration of the signal to noise ratio (SNR) related to the optical homodyne and heterodyne detection has been discussed previously [18]. The relative intensity noise (RIN) and phase noise of laser and other optical phase variations caused by the internal factors of the LDV and surrounding noises will restrict its minimum detectable capability. Compared with the homodyne detection, the heterodyne detection can provide a good performance in the frequency band less than 1 kHz at the expense of a much more complicated optical implementation and the extra signal demodulation processing for the IF signal. However, the serious IF crosstalk signal will impair the heterodyne LDV performance, and substituting the fiber circulator with a polarization prism can eliminate this crosstalk to a certain extent [19].

In recent years, the growing maturity of the LDV makes various types of LDVs commercially available. Several types of multi-channel and multipoint LDVs have been well established; besides, the continuous scanning LDV is investigated to measure the out-of-plane vibration of a structure surface [20]. However, the frequency bandwidth should be taken into account when realizing the LDV-based underwater acousto-optic communication. Under the hydrodynamic air–water interface, the tiny vibration caused by the underwater sound source is amplitude modulated by the water surface wave motion, and the movement of the water surface is at the level of cm/s or above. In order to obtain the transmitted frequency of the underwater sound source, the water fluctuation must be detected without any missing [21]. Besides, for the nondestructive testing (NDT) with the scanning LDV, it is time-consuming and easy to lose focus during the scanning situation [22,23].

Whatever the single-point LDV, the multi-point LDV or the scanning LDV, the performance of this small-amplitude vibration measurement requires that the spatial resolution or detection sensitivity be as sensitive as possible. Reducing the noise floor level of the LDV is an effective way to improve the performance of the LDV. In order to promote the detection sensitivity, many analyses related to the measurement noise have been done [24]. The influence of the internal parasitic reflections of the LDV is analyzed in Ref [25]. When measuring the rotational angular displacement with dual LDVs, Ref [26] improves the measurement accuracy to 0.0088° after analyzing the influence of the light intensity non-uniformity error, the systematic defect error, the synchronization error, and the sampling frequency-caused error. Besides the analysis of SNR of the LDV, diverse optical configurations and signal processing methods have been reported in the scientific literature and by commercial manufacturers. With the birefringent dual-frequency LDV and a new signal-processing scheme based on a digital signal processor lock-in amplifier and a low-frequency demodulation algorithm, the average velocity resolution of this dual-frequency LDV is improved from 0.31 to 0.028 mm/s due to the characteristics of good directionality, low speckle noise, and good coherence [27]. By using the 1550 nm all-fiber pulsed LDV and a new digital range gated signal processing method, the cochannel interference can be eliminated, and the SNR of the demodulated signal is improved consequently [28]. As the spectrum leakage and fence effect decrease the measurement precision of the LDV significantly, the trispectral interpolation of the Nuttall window is proposed for the LDV signal processing [29]. For the four-channel heterodyne LDV developed in Ref [30,31], a new calibration method based on the Bessel function of the first kind is introduced to improve the measurement resolution. The minimum detectable displacement of this LDV is up to 0.7 nm (RMS) at a bandwidth of 90 kHz and the vibration velocity can reach 3.8 m/s. Recently, Ref. [32] employs the phase multiplication to enhance the LDV measurement resolution, and the minimum detectable displacement of 72 nm with an error range of ±14 nm is achieved through direct counting of interference fringes.

In view of our previous research, it was observed that the match and the balance between the local oscillator (LO) laser beam and the transmitted laser beam is a fundamental and critical guarantee for achieving the highly sensitive experiment results. Insufficient control may produce associated noise and measurement error. In addition, referring to the acousto-optic detection for the underwater sound, the infrared radiation laser is more advantageous than other lasers. Besides, the in-air platforms give a real demand that the LDV should be able to present a non-contact and fast response with a high performance as well as a simple and easy-implementable structure.

In this letter, a compact cost-effective eye-safe wide-band high-sensitivity homodyne LDV with a self-correction ability of nonorthogonality and nonlinearity is designed. With a piece of thin glass pasted on the ring-shaped piezoceramic transducer (PZT), the minimum resolvable velocity resolution and the frequency response of this self-made homodyne LDV are investigated. With the compensation of the optical path on the LO laser arm, the system background noise is able to be kept at the same level with an increase in the detection distance. Compared with the results obtained with the air-water interface under the hydrostatic water surface in the anechoic tank, the detection capabilities acquired with the vibrating glass are discussed.

## 2. Homodyne LDV Instrument and Principle

**Figure 1:**
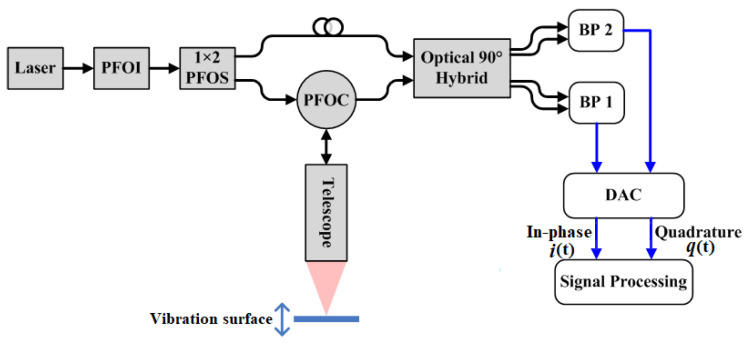
Schematic layout of the homodyne LDV.

Due to the rapid progress in the compact solid-state and fiber laser technology, the laser sources get an excellent opportunity, and components operating at eye-safe wavelength range are commercially available. In this case, it is possible to build more compact LDVs with small power consumption and low-cost fiber optical components. The schematic layout of the LDV based on the homodyne detection is shown in Figure 1. The laser operating at a wavelength *λ* of 1550 nm is the single-mode continuous-wave linearly polarizing semiconductor laser with an output power of 5 mW and a spectral line width of 1 kHz. After the single-mode polarizing fiber optical isolator (PFOI), the laser beam was split into two parts by a 1 × 2 single-mode polarizing fiber optical splitter (PFOS). To be specific, the first part acts as the LO laser beam while the second part plays the role of the transmitted laser beam. Through a single-mode polarizing fiber optical circulator (PFOC) and a telescope consisting of an aspherical lens with the aperture of 50 mm and the focal length of 200 mm, the transmitted laser beam was directed normally and focuses on the vibration surface. Meanwhile, the returned optical signal was collected with the same telescope and mixed with the LO laser beam in the single-mode polarizing optical 2 × 4 90° hybrid. Instead of the traditional electrical method realizing the phase difference of 90°, the optical 2 × 4 90° hybrid here can eliminate the random phase drift caused by the heat from the chips and guarantee a stable phase difference. Then, the mixed optical signals were detected by the balanced photoreceiver 1 (BP1) and balanced photoreceiver 2 (BP2) in order to reduce the influence of the common noise as much as possible. The outputs of BP1 and BP2 were frequency modulated signals and the modulation frequency was the Doppler shift frequency caused by the vibration of the detected surface. Subsequently, the two output signals were sampled by a dual-channel high-speed data acquisition card (DAC), and then undertook signal processing in the computer. In order to make the system set-up and the alignment more flexible and reliable, the fiber optical components were used. In addition to that, as the phase changing comes from the optical signal propagation difference between two arms of this homodyne LDV, an easy and promising implement idea is to add a certain length fiber onto the LO laser beam arm. Therefore, the local noise of this LDV can be kept at the same level even if the detection distance increases.

Theoretically, the phase difference between the outputs of BP1 and BP2 was 90°. Whereas suffering the minor phase imbalance of the two beams, imperfect phase bias offset of the optical 90° hybrid, and other factors in practice, there are the quadrature imbalance and other nonlinearity that can decrease the phase measuring accuracy to a great extent. Therefore, signal processing falls into two steps. In the first step, the nonorthogonal correction was to carry out the correction algorithm to modify the quadrature phase shift error and other systematic phase imbalance errors; in the second step, the discrimination algorithm tended to obtain the interested vibration parameters of the vibrating surface.

The outputs of BP1 and BP2 are described as the in-phase signal *i(t)* and the quadrature signal *q(t)*, shown in Equations (1)–(3). Due to the mismatch of quadrature and other nonlinearity under the actual operating condition, there was some error on the 90° phase difference between *i(t)* and *q(t)*. Consequently, *i(t)* and *q(t)* constitute one ellipse depicted in Equation (4).

(1) $$i\left(t\right)=R\sqrt{P_{S}\cdot P_{LO}}cos\left(\Delta \varphi \right)=I_{1}+I_{2}cos\left(\Delta \varphi \right)$$

(2) $$q\left(t\right)=R\sqrt{P_{S}\cdot P_{LO}}sin\left(\Delta \varphi +\delta \right)=Q_{1}+Q_{2}cos\left(\Delta \varphi +\delta -\pi /2\right)$$

(3) $$\Delta \varphi =2\pi f_{d}\left(t\right)t$$

(4) $$\frac{{\left[i\left(t\right)-I_{1}\right]}^{2}}{I_{2}^{2}}+\frac{{\left[q\left(t\right)-Q_{1}\right]}^{2}}{Q_{2}^{2}}-\frac{2\left[i\left(t\right)-I_{1}\right]\left[q\left(t\right)-Q_{1}\right]sin\delta }{I_{2}\cdot Q_{2}}=cos^{2}\delta$$

where *R* is the BPs’ responsivity; *δ* refers to the random phase difference caused by the LO laser beam, the transmitted laser beam, and other non-ideal factors; *P*_S_ and *P*_LO_ represent the average powers of the returned optical signal and the LO laser beam at the input of the optical 2 × 4 90° hybrid, and *f_d_(t)* denotes the laser Doppler frequency shift introduced by the moving vibration surface.

**Figure 2:**
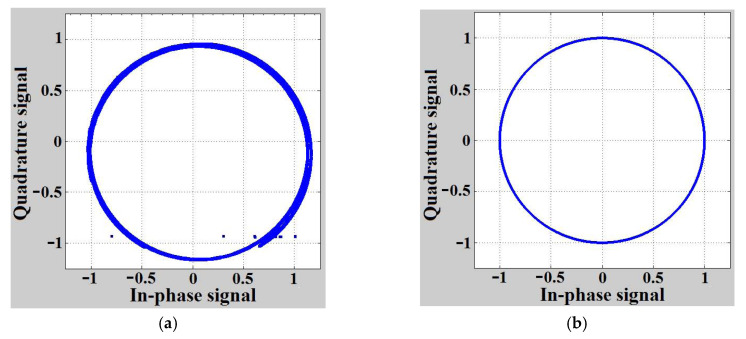
(**a**) The original set of in-phase signal *i(t)* and quadrature signal *q(t)* from BP1 and BP2; (**b**) the corrected signals *i_1_(t)* and *q_1_(t)* after the nonorthogonal correction algorithm.

With the analog-to-digital conversion, a series of discrete in-phase/quadrature signals *i(m)*/*q(m)* were obtained correspondingly, and fit an elliptical equation. Based on the method of the least-squares estimator [33], by employing the coefficients of this elliptical equation, the initial *i(t)* and *q(t)* were expressed. Following the least squares estimator, an algorithm of circle fitting [34] was applied in order to make the in-phase and quadrature signals orthogonal completely. In Figure 2, the in-phase and quadrature signals before and after the correction for nonorthogonality and nonlinearity are depicted. The original outputs of BP1 and BP2 are displayed in Figure 2a and are close to the quadrature with each other. By means of the correction algorithm of the least-squares estimator and the circle fitting, the final in-phase and quadrature signals *i_1_(t)* and *q_1_(t)* are shown in Figure 2b, and they are orthogonal and constitute a perfect unit circle whose coordinates are (0, 0).

(5) $$i_{1}\left(t\right)=\cos\left(\Delta \varphi \right)=\cos\left[2\pi f_{d}\left(t\right)t\right]$$

(6) $$q_{1}\left(t\right)=\sin\left(\Delta \varphi \right)=\sin\left[2\pi f_{d}\left(t\right)t\right]$$

After the correction mentioned above, with *i_1_(t)* and *q_1_(t)*, the discrimination algorithm abstracting the laser Doppler frequency was carried out. The final output *u_OUT_(t)* of the signal processing is shown as Equation (7).

(7) $$u_{OUT}\left(t\right)=\frac{\frac{d\left[q_{1}\left(t\right)\right]}{dt}\cdot \left[i_{1}\left(t\right)\right]-\frac{d\left[i_{1}\left(t\right)\right]}{dt}\cdot \left[q_{1}\left(t\right)\right]}{{\left[i_{1}\left(t\right)\right]}^{2}+{\left[q_{1}\left(t\right)\right]}^{2}}=2\pi f_{d}\left(t\right)$$

Therefore, the minimum resolvable velocity or velocity resolution *v_min_(t)* of the vibration surface in the direction of the incident laser beam corresponds to the minimum resolvable Doppler frequency shift $f_{d_{min}}\left(t\right)$ and is denoted as Equation (8). Thus, with the help of $f_{d_{min}}\left(t\right)$, the minimum resolvable velocity *v*_min_*(t)* can be achieved.

(8) $$v_{min}\left(t\right)=\frac{\lambda }{2}f_{d_{min}}\left(t\right)$$

## 3. Calibration Experiments and Results

### 3.1. Calibration Tests of Detection Capability with Glass and PZT

**Figure 3:**
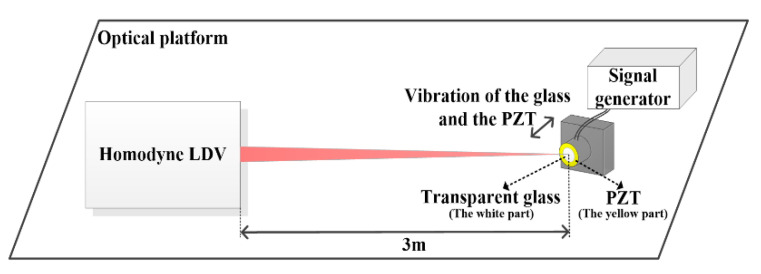
The calibration test setup for the detection capabilities of the LDV.

In order to verify the detection capabilities of the designed homodyne LDV and compare the test results with the theoretical vibration values of the detected surface, the calibration tests were conducted under room temperature and normal conditions with the setup shown in Figure 3. The detected vibrating target included a ring-shaped PZT (the yellow part) and a piece of same-size thin transparent glass (the white part) stuck on its surface. The glass and the PZT were mounted at a fixed distance of 3 m away from the homodyne LDV and exhibit a vibration totally depending on PZT response to its driving signal output from the signal generator. Here, the driving signal was a repeated pulse-modulated sine function with the sine function frequency of 4 kHz, the pulse modulation period of 1 s, and the duty cycle of 50:50, respectively. Correspondingly, the vibration of the glass and the PZT is a simple harmonic vibration, and the theoretical vibration displacement *s(t)* and velocity *v(t)* can be expressed as Equations (9) and (10).

(9) s(t)=σAsin(2πft)

(10) v(t)=2πfσAcos(2πft)

where *σ* is the strain coefficient of the PZT, and it is 5 nm/V; *A* and *f* are the amplitude and frequency of the sine function part belonging to the driving signal which were equal to 0.5 mV and 4 kHz, respectively. In this case, the theoretical maximum vibration velocity of the glass and the PZT was 62.8 nm/s.

**Figure 4:**
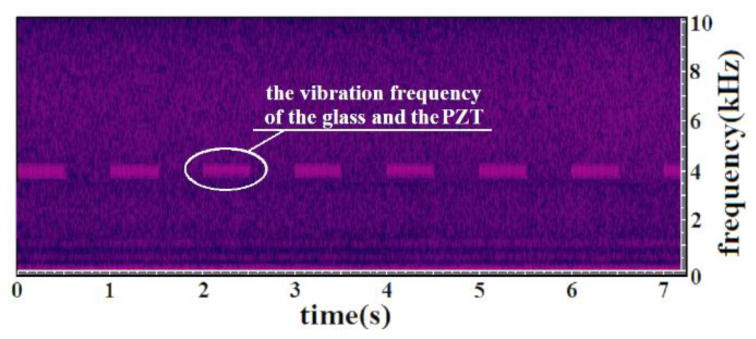
Time–frequency analysis of the homodyne LDV output.

**Figure 5:**
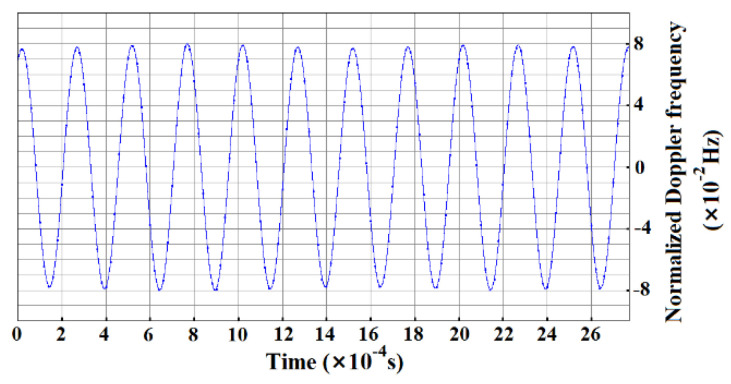
Time-domain waveform of the homodyne LDV output at 4 kHz.

Figure 4 is a time-frequency diagram of the homodyne LDV output. The 4 kHz signal represents the vibration frequency of the glass and the PZT, while its lasting time is consistent with that of the driving signal which is 0.5 s. For a selected small segment (0 to 2.6 ms) of the signal in Figure 4, the corresponding time-domain waveform is displayed in Figure 5, and its longitudinal coordinate denotes the normalized Doppler frequency shift. Consequently, the minimum distinguishable Doppler frequency shift of the homodyne LDV is about 0.08 Hz and its corresponding minimum detectable velocity *v*_min_ reaches 62 nm/s according to Equation (8). Compared with the theoretical vibration velocity of 62.8 nm/s, the measuring uncertainty is about 1.28%. The velocity resolution of the designed LDV is inherently frequency sensitive, and depends both on the accuracy of the Doppler frequency shift measurement and the noise and/or drift during this measurement. Therefore, not only the background noise or SNR of the LDV but also the surrounding around the LDV influences the measurement accuracy. In order to further improve the measurement accuracy and other detection performance of this LDV, it is necessary to place the LDV on the vibration-isolating platform and reduce the effect of air turbulence as much as possible besides optimizing the structure and the parameters of the LDV. Furthermore, it can be observed that there are frequency disturbances of 1 kHz and 500 Hz in Figure 4, and the time-domain waveform is fluctuating a little with the time in Figure 5, which mostly results from the following two major aspects, namely the surrounding noises, disturbances, and other unexpected vibration except for the undergoing detected vibration, and the amplitude instability from the homodyne LDV, such as the drift of laser intensity and the voltage amplitude fluctuation of photoreceivers.

The LDV is a vibration or velocity-sensitive system. Each corrupt that can be effectively converted to the velocity or vibration will cause frequency interference to the final output correspondingly. Thus, besides the phase control by using the correction algorithms mentioned before, it is also critical to address the issue of keeping good amplitude stability for the high sensitivity.

When the working frequencies of the calibrated PZT are far away from its resonance frequency, based on Equations (9) and (10), it is obvious that the PZT vibration displacement is directly linear to the voltage of the given driving signal, and has no relationship with the frequency of the driving signal. However, the vibration velocity of the PZT was not only affected by the voltage but also influenced by the frequency of the driving signal. Therefore, on the critical state of the vibration detection, the minimum detectable capabilities of this designed homodyne LDV were obtained. Its minimum detectable displacement can be up to 2.468 pm at 4 kHz. Meanwhile, when the driving signal frequency is lower than the resonance point of the PZT, the minimum detectable velocity will scale with the frequencies of the driving signals. Depending on the minimum detectable velocity *v*_min_ at 4 kHz, the velocity resolutions at other frequency bands can be calculated one by one.

**Figure 6:**
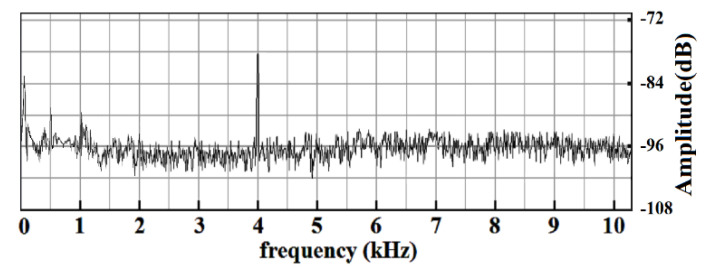
Amplitude spectrum of the homodyne LDV output with the vibrating glass and PZT.

With the help of the glass and the PZT, it was meaningful to evaluate the homodyne LDV performances (frequency response, local noise, minimum detectable capabilities, and others) for the remote voice acquirement and the NDT of composite materials. Considering these two applications, the LDV spectrum range was studied from 0 to 10 kHz. When the system minimum detectable velocity *v*_min_ reaches 62 nm/s, by using its corresponding amplitude spectrum (Figure 6), there was an obvious peak that can be observed at 4 kHz, and the SNR was approximately 17 dB.

In contrast with the heterodyne LDV [35], it is meaningful that the homodyne LDV has removed the disturbance in the frequency of 4 and 8 kHz significantly resulting from the in-phase and quadrature demodulation circuit centering at the frequency of 55 MHz. In terms of the frequency response, the homodyne LDV was consistent with that of the driving signal to the PZT. Its frequency error was no more than ±1 Hz. In addition, the homodyne detection mode achieved the same background noise level after 3 kHz as that of the heterodyne detection mode with a reliable, simple, and cost-effective structure.

### 3.2. Calibration Tests of Detection Capability with the Hydrostatic Air-Water Surface under the Anechoic Tank

One profound application of the LDV is the acousto-optic communication which is capable of receiving the acoustic signal without any mechanical contact. As for the acousto-optic communication under the actual water situation, the minimum detectable sound pressure level (SPL) of the LDV is the critical factor that determines the fundamental detection limit of the whole LDV-based sensing system.

**Figure 7:**
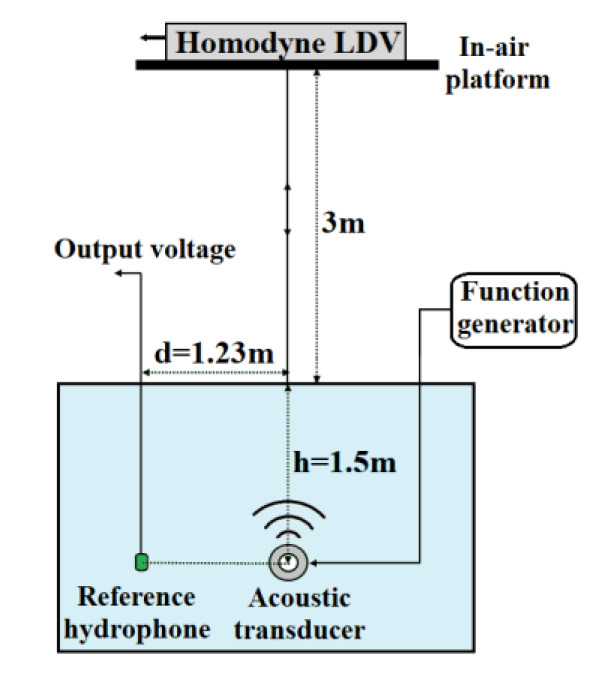
The setup of calibration tests of detection capability with the air–water surface under the anechoic tank.

For specifying the minimum detectable SPL of the designed homodyne LDV, the minimum detectable SPL calibration tests were performed under the anechoic tank at the National Defense Underwater Acoustics Calibration Laboratory. The experiment setup was similar to that mentioned in the previous report [28]. The homodyne LDV was located on the in-air platform, and the distance between the transmitted telescope of the LDV and the water surface was 3 m (Figure 7). The underwater acoustic transmitter was just directly below the detection point of the LDV, and it was 1.5 m away from the water surface. Using one function generator, the underwater acoustic transmitter was set to emit a 2 ms acoustic pulse of sinusoidal signals repeating every 0.5 s. During the test, the underwater acoustic signal was observed using a ceramic-based reference hydrophone TC4019, 1.23 m away from the acoustic transmitter. The output voltage *u_OC_* of the reference hydrophone TC4019 was the calibrating signal, and it demonstrated the minimum detectable capability of the LDV. For the tested sinusoidal signal, the frequencies were chosen from 6 to 200 kHz. In this way, not only can the surrounding disturbance distributing in the low-frequency band be prevented effectively, but it can also meet the TC4019 frequency bandwidth limitation of 200 kHz.

**Figure 8:**
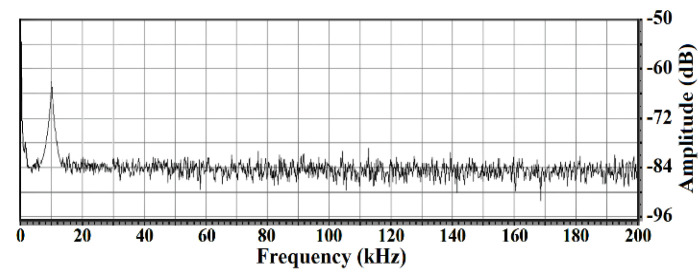
Amplitude spectrum of the homodyne LDV output with the air–water surface.

Through a series of calibration tests, the demodulated output signals were obtained with the help of the homodyne LDV. As the amplitudes of the function generator reduced, the output signal amplitudes of the LDV decreased at the same time. When the output signal of the LDV became just indistinguishable from the background noise, the minimum detectable SPL and the amplitude spectrum could be acquired based on the TC4019 output voltage *u_OC_*. Under the hydrostatic surfaces, the corresponding minimum detectable SPLs of the homodyne LDV at different frequency bands were compared. After that, when the transmitted acoustic pulse of the sinusoidal signal was at 10 kHz, the LDV reached its detection limit that the minimum detectable SPL was as low as 0.12 Pa. At this time, the *u_OC_* was about 20 mV measured by an oscilloscope. Figure 8 shows the Fourier transforms of the LDV output when the detected acoustic pulse was at 10 kHz. It was clear that the SNR was almost 21 dB when the LDV reached the level of the minimum detectable SPL which was a preferable frequency band for the realization of the acousto-optic communication. In view of the FM communication, the designed homodyne LDV was still featured with good extendibility because the LDV had a wide frequency bandwidth and a large dynamic range.

## 4. Differences between Background Noise of the LDV with Glass and that with the Air–Water Surface

Only with different kinds of detected surfaces, the glass and the air–water surface, were there some differences in the same homodyne LDV response performances.

When the detected surface was the glass whose vibration was caused by the PZT and its corresponding driving signals, the noise distribution shown in Figure 6 was in line with the same conclusion from Figure 4, and it mainly occupied the frequency range under 1.5 kHz. Subjected to the characteristic of the photoreceivers and the inevitable and undesirable vibrations in the measurement environment, this spectral distribution in this frequency range was mainly affected by the 1/*f* noise. However, the local noise spectral distribution above 3 kHz was flat and was maintained at –96 dB which is an effective guarantee for small-amplitude vibration testing in this frequency range. In this case, it is a good choice for the NDT of composite materials. In addition, as for the remote voice acquirement, it is necessary to suppress or remove the fixed disturbance of 500 Hz and 1 kHz within the audio range so as not to affect the voice signal measurement. In the case of the acousto-optic communication, the detected hydrostatic air-water surface remarkably leads to performance deterioration in the background noise of the LDV. The local noise spectral distribution appears at −84 dB from 2 to 200 kHz, and besides, the noise below 2 kHz badly affects the detection of the underwater sound in the low-frequency band.

In the calibration experiments, the minimum detectable capabilities were both obtained when the output signals of the LDV were indistinguishable from the background noise. When the LDV reached its detection limits with the detected vibrating surface of the glass and the PZT, the driving signal to PZT was 0.5 mV. At this time, the background noise of the LDV is −96 dB and the SNR was 17 dB. Therefore, the signal was −79 dB. For the LDV-based acousto-optic communication, the detected vibrating surface was the hydrostatic air–water interface, and the output voltage *u_OC_* of the TC4019 was the calibrating signal demonstrating the minimum detectable capability of the LDV. When the LDV reached its detection limits, the *u_OC_* was 20 mV. Meanwhile, the background noise of the LDV was −84 dB, and the SNR is 21 dB. Therefore, the signal was −63 dB. For the same LDV and nearly the same experimental conditions (such as the detection distance, the reflection coefficients of the glass and the water surface, the lab environment, and so on), the different levels of background noise of this designed homodyne LDV were mainly affected by the structural features of these two kinds of detected vibrating surfaces, and the total difference can be equivalently expressed as 16 dB in the power spectrum. Due to the mismatch of the acoustic impedance between the air and the water, the air–water interface is the underwater sound pressure releasing surface, and vibrates at the same frequency as the incident acoustic fields when the sound wave travels to the water surface. However, the air–water interface’s vibration response to the sound was less than that of the glass. In addition, the hydrostatic air–water surface is the structure of liquid whose rigidity is weak and, therefore, induces much more speckle noise than that of glass. Consequently, the vibration response performance to the acoustic signal in the LDV-based acousto-optic communication with the detected air–water surface differs from that in LDV-based voice signal detection with the detected glass or the similar solid media. The SPL of the underwater sound source needs to be stronger in order to compensate the worsened background noise caused by the hydrostatic air-water interface. The amplitude relationship between the driving signal to the PZT and the output voltage *u_OC_* of the TC4019 consists with the variation of the background noise of these two kinds of vibrating surfaces. Furthermore, it is obvious that the SNR of the LDV-based sensing system will rapidly deteriorate under the real water surface conditions suffering from the water motion and the wind disturbance.

In respect to the frequency response range, whatever the glass or the air–water surface, the homodyne LDV was capable of providing a wide frequency bandwidth and has a good consistency with that of the incident soundwave. The frequency error is no more than ±1 Hz, and the time delay is about 49 ns under the ideal condition. In addition, assuming the sampling frequency of DAC is 2.56 times the detected laser Doppler frequency shift *f_d_(t)* based on the traditional sampling theory-Nyquist sampling theorem, the detectable maximum vibration velocity of the designed LDV can be up to 7.57 m/s. Therefore, this homodyne LDV can satisfy the requirement for vibration measurement in most cases.

## 5. Conclusions

Aiming for the acousto-optic sensing, the voice signal detection and the future NDT for the composite material, an all-fiber homodyne LDV is demonstrated. Referring to the least-squares method, with the signal processing involving the correction of the quadrature imbalance and other nonlinearity, the orthogonal state between the in-phase and quadrature signals can be realized. In the calibration experiment, with the laser power of 5 mW, the telescope aperture of 50 mm, the detection distance of 3 m, and a piece of thin glass pasted on the ring-shaped PZT whose driving signal is 0.5 mV at 4 kHz, the designed homodyne LDV shows good performance with extreme accuracy and sensitivity. The measured vibration velocity and vibration displacement can reach 62 nm/s and 2.468 pm respectively. Thus, the vibration values of the glass and the PZT obtained from the experiments differ from the theoretically calculated ones within 1.28%. Compared with the heterodyne detection mode, there was no disturbance at the frequency of 4 and 8 kHz, and the background noise and the SNR from 3 to 10 kHz are able to be at the same level as that of heterodyne LDV. For the NDT of composite material, it is necessary to develop the multi-point LDV to meet the engineering requirements in terms of efficiency, real-time, and rapidness. There is no doubt that the performance of the multi-point LDV relies on the single point LDV. The detection sensitivity of the single point LDV is overarching consideration to build one practical multi-point LDV so that the weak signal can be picked up from the surroundings.

For the detection or communication with the underwater sound source, the requirements are more specific and demanding than those of the normal industrial applications, and the minimum detectable SPL is also the fundamental factor. Besides, the tiny vibration caused by the underwater sound is mixed with the obvious fluctuation of the water surface (up to the level of m/s) which means the high-speed data acquisition must be applied. For the purpose of these, the proposed LDV is both with a good minimum detectable SPL of 0.12 Pa and a large frequency response bandwidth of 25 MS/s per channel. However, when the LDV is used for the acousto-optic communication under the hydrostatic air-water interface, the background noise of the same LDV is approximately 16 dB worse than that obtained with the transparent glass and the PZT, which is mainly caused by the characteristics of the detected air–water interface.

To summarize, for the unidirectional vibration measurements, such as the LDV-based voice signal detection and LDV-based NDT of composite materials, this homodyne LDV has more promising potential to offer a good choice when concerning pointing a low-power laser beam at the remote target and obtaining the vibration information in a cost-effective, compact, precise, and high-sensitivity way. In our following research, the control of the amplitude stability or the approach to eliminating its influence will be further studied. Meanwhile, a series of associated acousto-optic practical applications and the diagnostics for the composite material relying on this homodyne LDV will be drawn.
